# Effect of Ultrasound‐Stimulated Microbubbles and Hyperthermia on Tumor Vasculature of Breast Cancer Xenograft

**DOI:** 10.1002/jum.15950

**Published:** 2022-02-10

**Authors:** Deepa Sharma, Holliday Cartar, Karina Quiaoit, Niki Law, Anoja Giles, Gregory J. Czarnota

**Affiliations:** ^1^ Physical Sciences Sunnybrook Research Institute Toronto Ontario Canada; ^2^ Department of Radiation Oncology Sunnybrook Health Sciences Centre Toronto Ontario Canada; ^3^ Departments of Medical Biophysics, and Radiation Oncology University of Toronto Toronto Ontario Canada

**Keywords:** breast cancer, hyperthermia, microbubbles, ultrasound, vasculature

## Abstract

**Objective:**

The objective of the present study was to investigate the treatment effects of ultrasound‐stimulated microbubbles (USMB) and hyperthermia (HT) on breast tumor vasculature.

**Methods:**

Tumor‐bearing mice with breast cancer xenografts (MDA‐MB‐231), were exposed to different treatment conditions consisting of control (no treatment), USMB alone, HT alone, USMB with HT exposures of 10 and 50 minutes. Quantitative 3D Doppler ultrasound and photoacoustic imaging were used to detect tumor blood flow and oxygen saturation, respectively. In addition, histopathological analysis including TUNEL staining for cell death, and CD31 staining for the vessel count, was performed to complement the results of power Doppler and photoacoustic imaging.

**Results:**

Results demonstrated a decrease in tumor blood flow as well as oxygenation level following 50 minutes HT treatment either alone or combined with USMB. In contrast, 10 minutes HT alone or combined with USMB had minimal effects on blood flow and tumor oxygenation level. Treatment with HT for 50 minutes caused drops in tumor oxygenation, which were not evident with USMB treatment alone. Additionally, results revealed an increase in cell death after 10 minutes HT with or without USMB and a decrease in vessel count compared to control. Unlike previous studies which demonstrated synergistic treatment effects combining USMB with other modalities such as radiation or chemotherapy, USMB and HT effects were not synergistic in the present study.

**Conclusion:**

The results here demonstrated HT and USMB both alone or together resulted in a significant reduction in tumor blood flow, tumor oxygenation, and vessel count with observed increases in cell death response.

**Abbreviations**
HT: hyperthermia
MB: microbubbles
PBS: phosphate‐buffered saline
TNF: tumor necrosis factor
TRAIL: TNF‐related apoptosis‐inducing ligand
USMB: ultrasound‐stimulated microbubbles
VI: vascular index

Hyperthermia (HT) has been used for centuries for therapeutic benefits. The treatment involves heating the entire body or heating selective areas or tissues. It is a cancer treatment modality in which target tissue is exposed to a high temperature ranging from 40 to 48°C.^1^ It can be administered as local, regional, or whole‐body HT. Local HT incorporates a temperature greater than 43°C where a small area like a tumor is targeted. Regional HT on the other hand is used to treat large parts or areas of the body using a temperature range of 40–45°C. Whole‐body HT uses a temperature around 42°C to elevate the entire body temperature. HT is known to cause direct damage to cancerous cells while leaving normal tissues with no or minimal injury.^2^ The primary mechanism of HT works by the degradation and denaturation of various protein structures ultimately leading to DNA damage.^3^ Depending on the size and site of the tumor, the type of HT is selected and administered. HT is administered in conjugation with radiation therapy or chemotherapy. For local HT treatment, the heat might be applied internally using a wire or needle probe that is heated or microwave antennae and radiofrequency electrodes are used. External heat is applied using high‐frequency waves to target the tumor using a device that is connected from outside the body. The temperature ranges from 39 to 45°C that lasts for 30–60 minutes.^4^ Whole‐body HT is usually used to treat metastatic cancers. The application of Whole‐body HT is performed by immersing the body in a hot water bath or by using water heater blankets, or a thermal chamber. The temperature for Whole‐body HT usually ranges from 39.5 to 41.0°C lasting for 3–4 hours or by increasing the temperature to 42.0°C for 60 minutes.^1^ HT treatment session usually takes several days to weeks depending on the tumor size, type, and site.^4^

Over the past few years, significant advances in combining HT with ultrasound‐stimulated microbubbles (USMB) therapy has led to promising outcomes in several pre‐clinical studies.^5^, ^6^ Microbubbles (MB) are microscopic bubbles composed of a gas core smaller than 10 μm encapsulated in a lipid or protein shell.^7^ Ultrasound waves are used to stimulate MB causing oscillation, expansion, and finally rupture resulting in structural changes to nearby cell membranes.^8^ Therapy consisting of USMB in combination with HT has demonstrated a statistically significant increase in tumor cell death in vitro.^5^, ^6^ Similarly, several in vivo studies have also been conducted combining these two therapies to increase therapeutic efficacy.^9^, ^10^ Both treatment modalities on their own are known to target tumor vasculature resulting in prominent changes in tumor blood flow and tumor oxygen content.^11^, ^12^ This study investigated the combined therapeutic effect of USMB with HT to detect the changes in tumor vasculature using power Doppler ultrasound and photoacoustic imaging.

Power Doppler is a non‐invasive imaging technique that permits the detection of blood flow as well as enabling monitoring of any changes in the vasculature. Doppler ultrasound provides 3D imaging volumes that can be used to determine relative changes in vascularity however, it fails to detect the flow in smaller vessels which limits its uses in tumor vasculature studies. As such, it can only determine the flow signal in the vessels that are greater than 30 μm in diameter which might influence the overall signal detected^13^, ^14^ and is sensitive to artifact.^15^ Photoacoustic is another non‐invasive imaging method that enables the assessment of hemoglobin levels and oxygen saturation levels. It has been used to discriminate between vascular and pigmented lesions.^16^ Its uses have also been reported for breast vasculature visualization^17^ as well as to monitor structural and functional aspects of the brain depending upon changes in blood volume and oxygen consumption.^18^ Even though Doppler ultrasound is seen to be effective while detecting total blood volume with high flow speeds and flow detection in larger vessels, photoacoustic imaging can detect flow even in small vessels with low flow speeds and is independent of any noise or movement created during data acquisition.^19^

In the study here, power Doppler and photoacoustic imaging were used to determine the changes in tumor vasculature upon USMB and HT treatment. Tumor blood flow was detected using the VEVO 770 imaging system and the oxygen saturation was estimated using Visual Sonics 2100. Further to complement imaging data, histopathological staining was performed using deoxynucleotidyl transferase dUTP nick end labeling (TUNEL) for cell death and cluster of differentiation 31 (CD31) for vessel labeling.

## Methods and Materials

This manuscript is exempted from obtaining informed consent.

All experimental procedures were conducted in compliance with protocols (# 18‐395) approved by the Sunnybrook Health Science Centre Institutional Animal Care and Use Committee.

### MDA‐MB‐231 Cancer Cell Line

Human adenocarcinoma breast cell line (MDA‐MB‐231) was obtained from the American Type Culture Collection (ATCC, MD, USA). Cells were cultured in RPMI‐1640 medium (Wisent BioProducts) supplemented with 10% fetal bovine serum (FBS, Sigma‐Aldrich) plus 1% penicillin/streptomycin antibiotics (ThermoFisher Scientific). Cells were kept in the incubator at 37°C in 5% CO_2_ until confluent. For injection, cells were collected using 0.05% Trypsin‐EDTA (Wisent BioProducts) followed by centrifugation at 4°C for 10 minutes at 200*g*. Cells were further suspended in Mg+/Ca+ Dulbecco's phosphate‐buffered saline (DPBS) at a concentration of 5 × 10^6^.

### Animal Model

Animal handling was performed following the guidelines of the Canadian Council on Animal Care and approved protocols by the Sunnybrook Research Institute Institutional Animal Care and Use Committee. For experiments, female severe combined immunodeficiency (SCID)‐B17 mice obtained from Charles River Canada were used. Cell suspension (100 μl) was injected into the right hind leg of each mouse administered subcutaneously using a 27 gauge needle. After 4–6 weeks, once a tumor reached the size of 5–10 mm in diameter, animals were used for the experiments.

Prior to treatment, mice were anesthetized using oxygen ventilated isoflurane for induction followed by injection of ketamine (100 mg/kg) and xylazine (5 mg/kg)  administered intraperitoneally. For maintaining regular body temperature animals were kept under heating lamps and monitored visually.

For each treatment group, four or more than four animals were used in this study.

### Ultrasound and Microbubble Treatment

For treatment, 3% (v/v) MB stimulated at 570 kPa ultrasound pressure and two different HT times (10 and 50 minutes) were investigated.

Definity® microbubbles (Lantheus Medical Imaging, Inc., North Billerica, MA, USA) were shaken for 45 seconds at 3000 rpm for activation using a Vialmix® (Lantheus Medical Imaging, Inc., North Billerica, MA, USA). Each mouse was injected with MB administered intravenously using a 26 gauge tail vein catheter followed by immediate flush using 150 μl (0.2%) heparin/saline.

For ultrasound exposure mice were mounted in a custom‐designed plastic chamber which was partially submerged in 37°C degassed water. The ultrasound therapy system consisted of a 2.85 cm unfocused planar transducer with central frequency 500 kHz (ValpeyFisher Inc, MA, USA). A waveform generator (AWG520, Tektronix) was connected to a power amplifier with pulse/receiver (RPR4000, Ritec) and a digital acquisition system (Acquiris DC440/PXI8570, Agilent Technologies, Canada, Mississauga, ON, Canada). A micropositioning system was used for treatment setup. Tumors were exposed to a 16 cycle tone burst at 500 kHz with a 3 kHz pulse repetition frequency. The total treatment duration lasted for 5 minutes with a peak negative pressure of ultrasound 570 kPa applied that was calibrated with a hydrophone.^20^, ^21^, ^22^

### Hyperthermia Treatment

Hyperthermia treatment was performed 5 hours after USMB treatment using a 43°C water bath. The time interval between USMB and HT incorporated in this study is based on our previous finding that suggests a gap of 3–6 hours between two different modalities produces maximum treatment effects.^20^ Mice were mounted in custom‐designed tubes with several holes to act as air vents which were further attached to custom‐made plastic plates with Velcro. The entire setup was placed in a water bath in such a way as to fully submerge the animal tumor in water. Tumors were exposed to HT for 10 or 50 minutes. An image illustrating the HT setup is presented in Supplemental Figure 1. Details of HT procedure is discussed elsewhere.^21^, ^22^

### Immunohistochemistry

Tumors were excised 24 hours after treatment and fixed in 10% neutral buffered formalin at room temperature for 48 hours. Tumor specimens were later transferred to 70% ethanol. Tumors were embedded in paraffin blocks and 5‐μm sections were placed on glass slides for further staining. Samples were stained with TUNEL for cell death and for CD31 for vessel detection (Pathology Research Program, University Health Network, Toronto, ON, Canada). For analysis, images of tumor specimens were captured and digitized (Leica MZ FL III, Leica Microsystems, Concord, ON, Canada) at a magnification of 0.8× for TUNEL and 20× for CD31. For TUNEL analysis digitized image files were analyzed using an in‐house custom code developed in MATLAB.^22^ CD31 staining was used to determine the mean vessel count (CD31 labeling) for which 5 regions of interest (ROI) were randomly selected per tumor section and the viable blood vessels were counted and averaged. Histology images presented in Figures 3A and 4A were captured using TissueScope LE scanner, Huron Digital Pathology (Kaga Electronics, USA) at a magnification of 25×.

### Power Doppler Ultrasound Imaging

Total blood flow in tumors was detected 24 hours before and after treatments using a VEVO 770 imaging system (Visual Sonics, Toronto, Canada) coupled to a transducer with a central frequency of 25 MHz. An RMV‐710B transducer was used with a wall filter setting of 2.5 mm/s, a step size of 0.2 mm, a scan speed of 2 mm/s, and a gain of 20 dB. Images acquired were analyzed using MATLAB‐based in‐house software (MathWorks, Natick, MA, USA).^12^, ^20^, ^23^ ROI were selected using all frames which consisted of 20 or more than 20 frames per tumor and data were averaged to determine the vascular index (VI). Change per tumor was obtained by comparing VI from before and after the treated animals.^20^, ^23^, ^24^

### Photoacoustic Imaging

Blood oxygen saturation was detected 24 hours before and after treatment using a photoacoustic imaging system. The data were obtained using Visual Sonics 2100 VEVO LAZR system (Visual Sonics, Toronto, Canada) and an 18 MHz central frequency transducer LZ‐250. The photoacoustic signal was determined using a gain of 51 dB. The level of oxygenated and deoxygenated hemoglobin was obtained from data using two optical wavelengths, 850 and 750 nm, respectively, using Visual Sonics 2100 software as described previously.^24^ The analysis was performed by averaging all the selected ROI within the tumor. Data were compared from before and after the treatment conditions to obtain the change per tumor.

### Statistical Analyses

All the data were analyzed using one‐way ANOVA followed by Bonferroni's selected comparison test with GraphPad Prism (GraphPad Software, La Jolla, CA, USA). Each treatment condition was compared to the control and USMB condition where (α < .05) was considered significantly different.

## Results

### Effect of USMB and HT on Tumor Blood Flow

**Figure 1:**
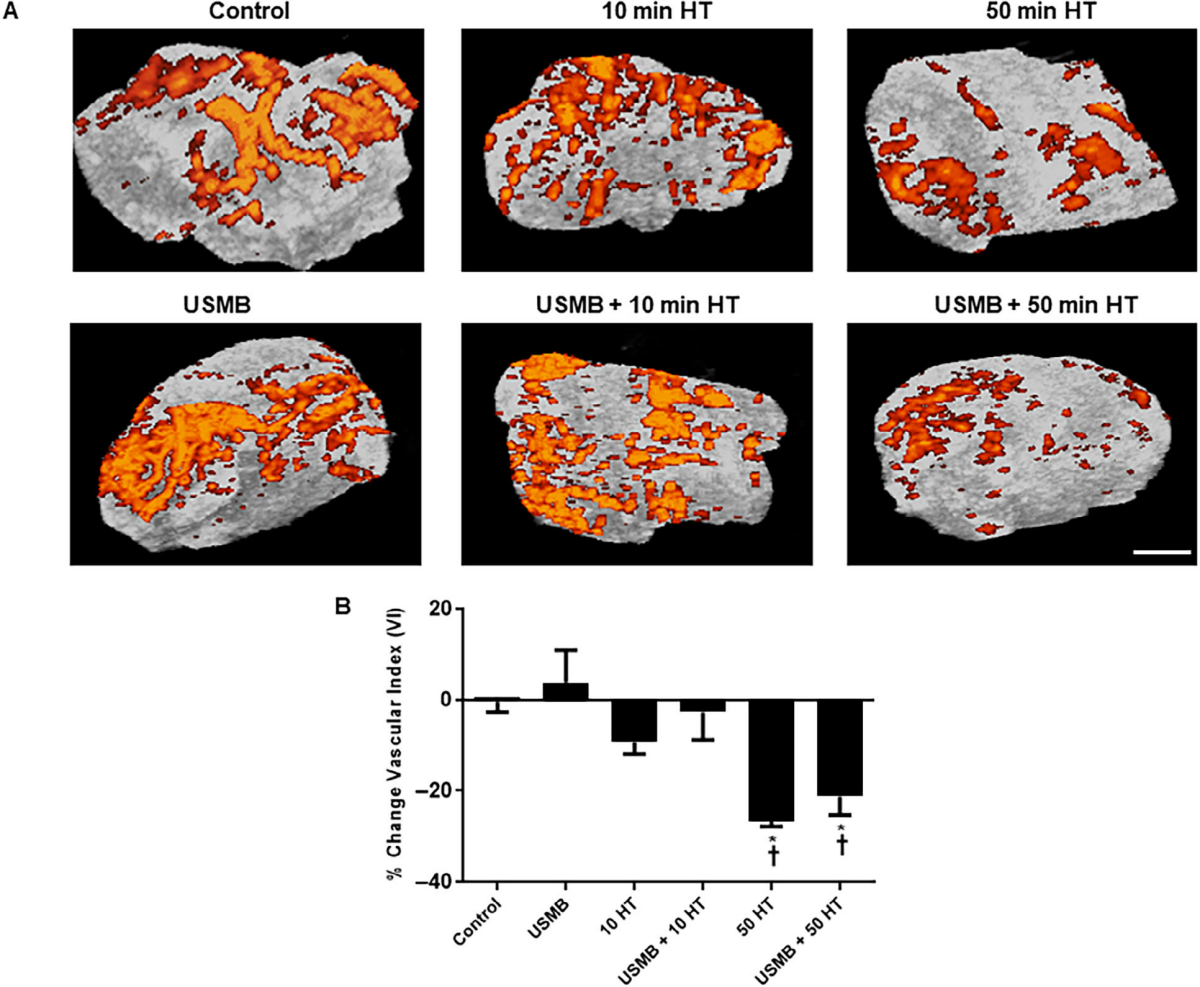
Changes in blood volume in MDA‐MB‐231 tumors after heating at 43°C for 10 and 50 minutes. **A**, Power Doppler images of xenograft tumor pre and post 24 hours after USMB and HT treatment. The magnification bar represents 1 mm. **B**, Quantification of power Doppler analyses revealed a significant decrease in blood flow with 50 minutes HT treatment either alone or combined with USMB compared to control or USMB alone. A statistical significance was determined using one‐way ANOVA followed by Bonferroni selected comparison test and the significance is indicated by *P < .05 for control and †*P* < .05 for USMB comparisons (HT, hyperthermia; USMB, ultrasound‐stimulated microbubbles).

Tumor vasculature changes were determined using power Doppler imaging of the tumors before and 24 hours after treatments. Representative images of tumor blood flow are presented in Figure 1A depicting an observed decrease in total blood flow with HT treatment. In Figure 1B, vascular index (VI) results determined from power Doppler analysis are presented. Results demonstrated a VI decrease of (−26.6 ± 1.2%) (mean ± SE) (*P* = .001) following HT treatment of 50 minutes alone. The combined treatment of USMB and 50 minutes HT resulted in reduced VI of (−21.2 ± 4.0%) (*P* = .0068). Both the treatments had a greater effect on tumor blood flow with a significant reduction compared to untreated control (−0.4 ± 2.3%). Additionally, a non‐significant decrease in VI was observed with HT treatment of 10 minutes alone (−9.3 ± 2.6%) (*P* = .1449) or HT combined with USMB (−2.7 ± 6.1%) (*P* = .7153) compared to control. A further comparison was also made between USMB‐only treated groups (3.8 ± 7.1%) with HT‐only exposed groups which revealed a statistically significant reduction in blood flow with 50 minutes HT alone (*P* = .0003) or combined treatment of USMB and 50 minutes HT (*P* = .002). However, 10 minutes HT alone or combined with USMB demonstrated no significant difference in blood flow compared to USMB‐only treatment.

### Effect of USMB and HT on Tumor Oxygen Saturation

**Figure 2:**
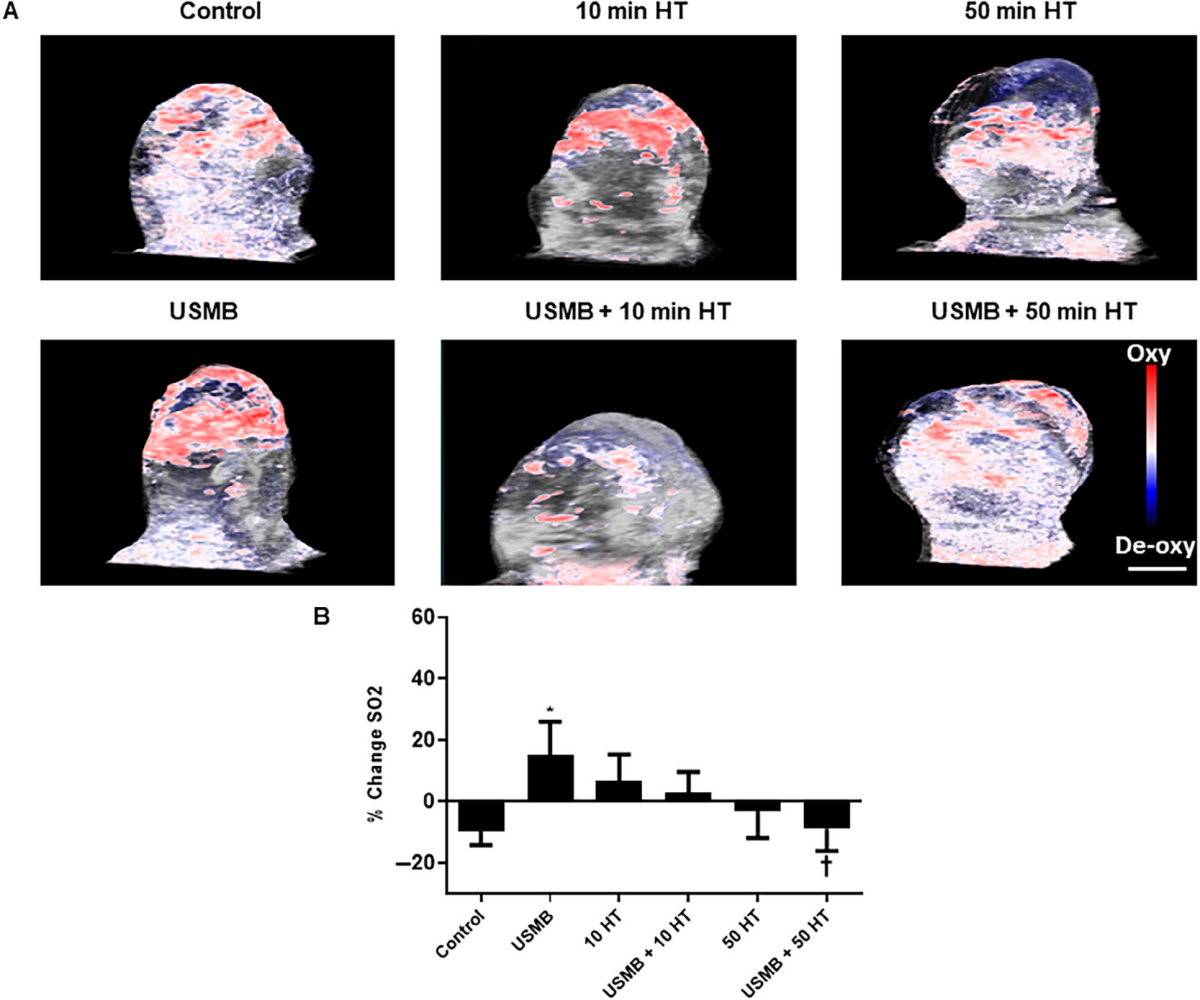
Photoacoustic analyses of treated MDA‐MB‐231 tumors. **A**, Photoacoustic post‐treatment images of MDA‐MB‐231 tumors obtained at 18 MHz indicate oxygen saturation with different treatments. The magnification bar represents 1 mm. **B**, Percent change in oxygen saturation levels 24 hours after USMB and HT treatment. Combined treatment of USMB with 50 minutes HT resulted in a significant decrease in oxygenation compared with USMB‐only treated animals while no significant effect was noticed with 10 minutes heat alone or combined with USMB. A statistical significance was determined using one‐way ANOVA followed by Bonferroni selected comparison test and the significance is indicated by *P < .05 for control and †*P* < .05 for USMB comparisons (HT, hyperthermia; USMB, ultrasound‐stimulated microbubbles).

Total oxygen saturation levels were acquired from photoacoustic imaging. Oxygenated and deoxygenated hemoglobin levels were determined using 850 and 750 nm laser wavelengths, respectively, during data acquisition. Figure 2A presents representative photoacoustic images of tumor oxygenation with various treatments. In Figure 2B quantification of tumor oxygenation demonstrated a significant increase in oxygen saturation level in the USMB‐only treated group (15.3 ± 10.7%) (mean ± SE) (*P* = .0208) compared to the control (−9.6 ± 4.6%). However, no detectable changes were observed with 10 minutes HT‐only (6.7 ± 8.6%) (*P* = .1229) or the combined treatment of USMB and 10 minutes HT (3.1 ± 6.6%) (*P* = .2103) comparing control. Moreover, 50 minutes HT alone (−2.9 ± 8.9%) (*P* = .5047) or combined with USMB (−8.7 ± 7.3) (*P* = .9289) showed no changes in oxygen saturation level compared to control. A further comparison was also carried out between USMB‐only treated groups and groups treated with HT alone or combined USMB and HT. The latter demonstrated a statistically significant reduction in oxygenation levels with the combined treatment of USMB and 50 minutes HT (*P* = .0344).

### USMB and HT Effect on Breast Tumor Cell Death

**Figure 3:**
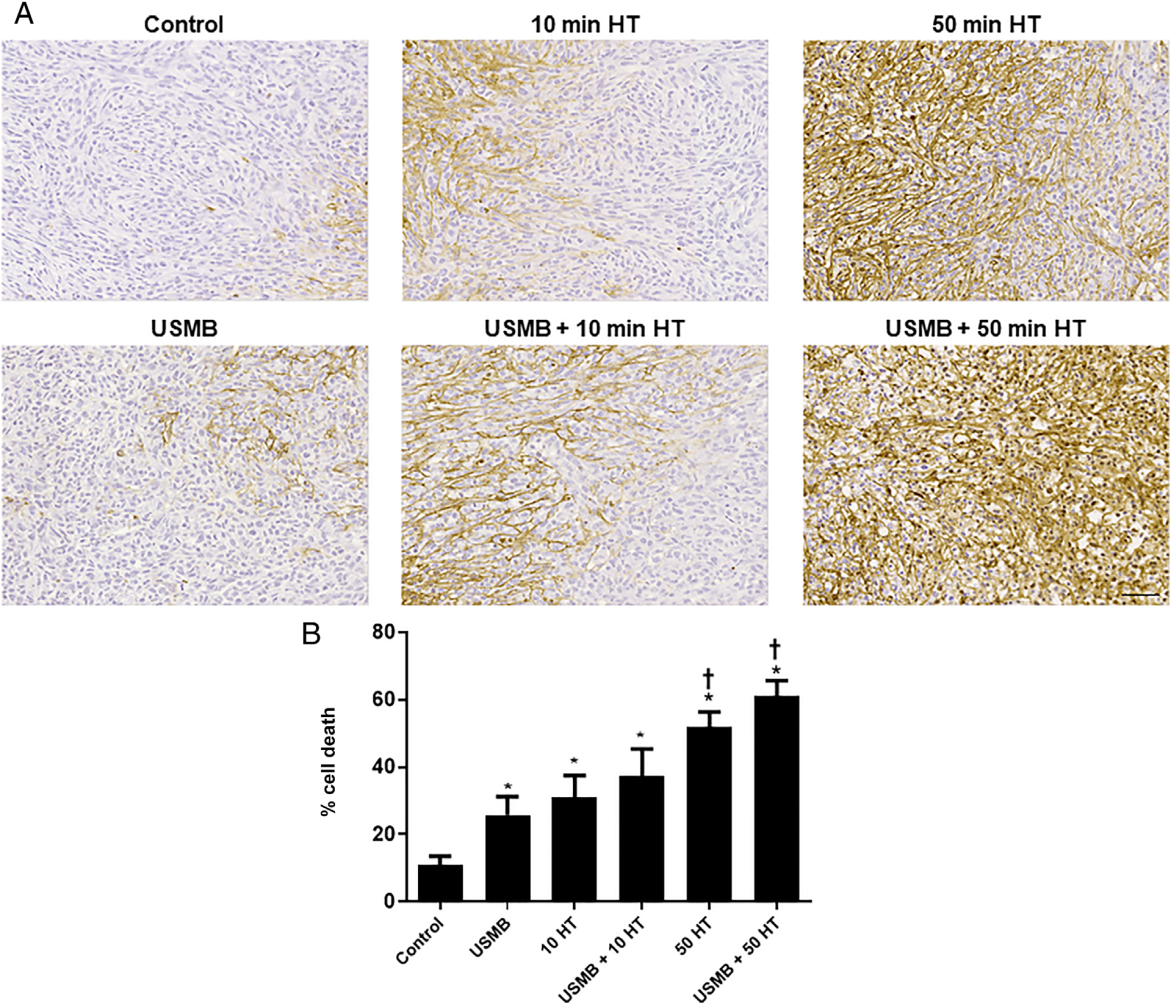
Histopathology, TUNEL of an MDA‐MB‐231 xenograft tumor. **A**, High magnification images of TUNEL staining of whole tumor sections treated with USMB, 10 minutes HT or 50 minutes HT with or without USMB. Brown color illustrates areas of cell death. The magnification bar represents 50 μm. **B**, Quantified analyses of TUNEL images, indicating an increased level of cell death with HT treatments compared to control. A significant increase in cell death with 50 minutes HT treatment either alone or combined with USMB was noticed compared to USMB‐only. Adapted from 22. A statistical significance was determined using one‐way ANOVA followed by Bonferroni selected comparison test and the significance is indicated by *P < .05 for control and †*P* < .05 for USMB comparisons (HT, hyperthermia; USMB, ultrasound‐stimulated microbubbles).

Investigation of tumor cell death was conducted using TUNEL staining. High magnification images of tumor sections demonstrating cell death are presented in Figure 3A. The images demonstrate fewer cell death regions (brown in color) in control (untreated) tumor sections with prominent increases in cell death with USMB and HT treatment. Quantitative analysis depicted in Figure 3B demonstrated significant cell death increase in tumor sections exposed to the treatment of 10 minutes HT alone (31.0 ± 6.5%) (mean ± SE) (*P* = .0073), or combined USMB and 10 minutes HT (37.1 ± 8.3%) (*P* = .0006) comparing control (10.9 ± 2.7%). Similarly, a prominent increase in cell death was noticed in groups receiving 50 minutes HT alone (51.8 ± 4.6%) (*P* < .0001) or combined treatment of USMB and 50 minutes HT (61.1 ± 4.6%) (*P* < .0001) compared to control groups.

### USMB and HT Effects on Vasculature Disruption of MDA‐MB‐231 Xenografts

**Figure 4:**
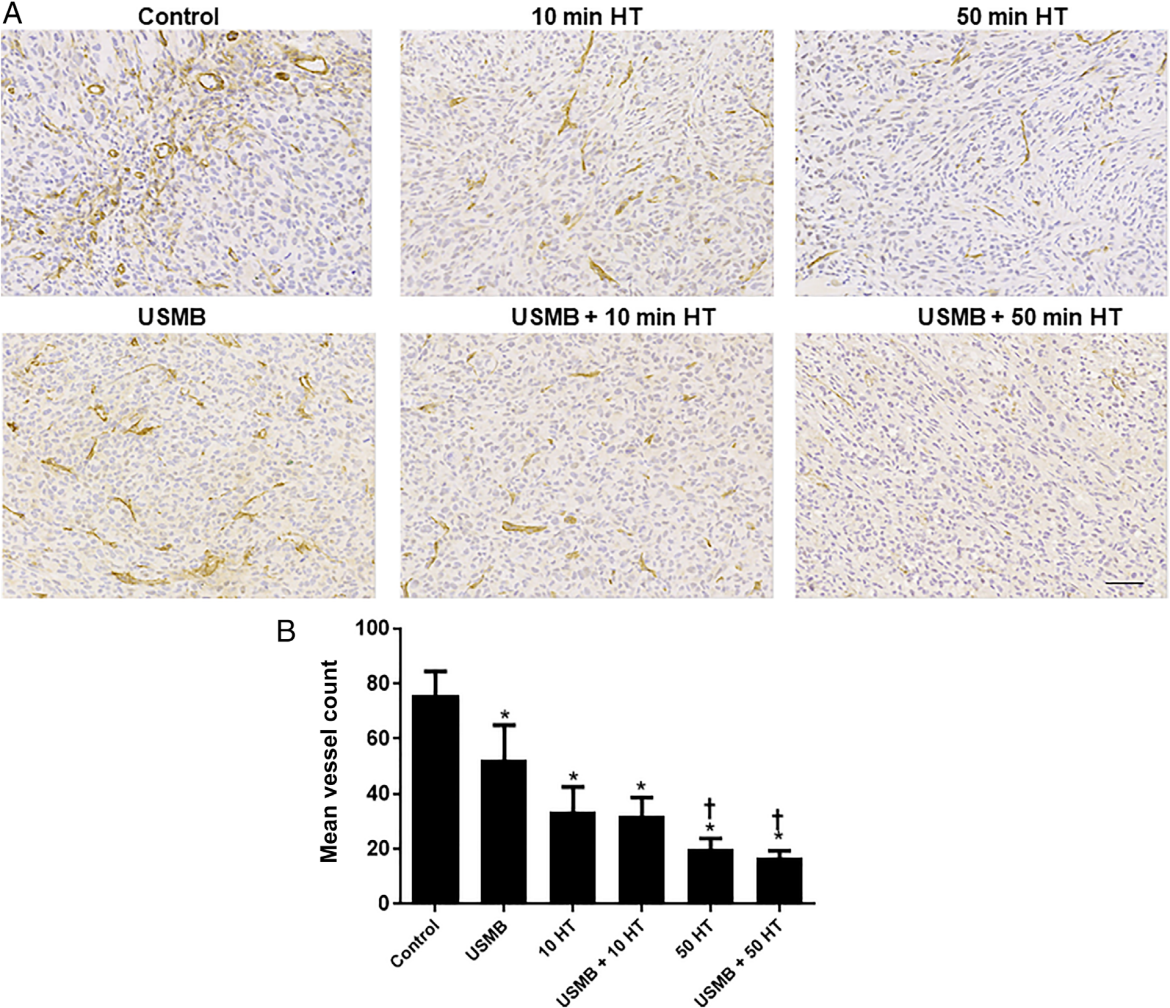
Vascular labeling of xenograft MDA‐MB‐231 tumor sections treated with USMB and HT: **A**, High magnification images of CD31 stained tumor sections with USMB and 10 or 50 minutes HT treatments. Tumor tissues of the control and USMB‐only treated group resulted in intact blood vessels (labeling for CD31) while exposure of 10 or 50 minutes HT resulted in significantly diminished stained blood vessels. The magnification bar represents 50 μm. **B**, Quantified CD31 staining for tumor tissue treated with USMB and HT. 10 and 50 minutes HT treatment revealed a significant reduction in the mean vessel count compared to the control or USMB‐only treated groups. Adapted from 22. A statistical significance was determined using one‐way ANOVA followed by Bonferroni selected comparison test and the significance is indicated by *P < .05 for control and †*P* < .05 for USMB comparisons (HT, hyperthermia; USMB, ultrasound‐stimulated microbubbles).

Treatment effects on vasculature were determined using CD31 immunohistochemistry. Figure 4A presents high magnification images of tumor sections treated with various treatment conditions. Results revealed that control groups and USMB‐only treated animals had relatively intact blood vessels whereas animals that were exposed to HT treatment of either 10 or 50 minutes demonstrated a diminishment of blood vessels. Quantitatively, compared to control animals (76.1 ± 8.4) (mean ± SE) treatment with 10 minutes HT alone resulted in a vessel count of (33.7 ± 8.8) (*P* = .0005) and treatment with combined USMB and 10 minutes HT resulted in vessel count of (32.1 ± 6.6) (*P* = .0003). Exposure to 50 minutes HT alone resulted in (20.1 ± 3.7) (*P* < .0001), whereas combined USMB and 50 minutes HT resulted in a diminishment in a vessel count of (16.8 ± 2.6) (*P* < .0001) compared to control animals (Figure 4B). Furthermore, when comparing the USMB‐only treated group (52.2 ± 12.9) versus 10 minutes HT alone (*P* = .1305) or combined USMB and 10 minutes HT (*P* = .1011), no significant reduction in stained blood vessels was observed. However, treatment with 50 minutes HT alone (*P* = .0102) or combined USMB and 50 minutes HT (*P* < .0001) showed prominent reductions in stained blood vessels compared to the USMB‐only treated group.

## Discussion

In the present study, Doppler ultrasound and photoacoustic imaging were used to monitor tumor vasculature responses to HT and ultrasound‐microbubble therapy. Power Doppler is a non‐invasive technique that has been used for detecting the flow responses in tumors upon implementation of various treatments.^12^, ^20^, ^24^, ^25^ Here, results demonstrated a decrease in blood flow with 10 minutes HT‐only or USMB + 10 minutes HT but the differences were not statistically significant compared to the control. However, blood flow was found to be significantly reduced in groups treated with 50 minutes HT‐only or combined treatment of USMB + 50 minutes HT compared to the control. Furthermore, a comparison made between USMB‐only treated groups to 10 minutes HT‐only or combined USMB + 10 minutes HT revealed no significant blood flow differences. However, animals that were exposed to either 50 minutes HT‐only or combined USMB + 50 minutes HT showed a prominent decrease in blood flow comparing USMB‐only. The findings here are partially in line with previous different work that demonstrated that the combination of USMB with radiation therapy results in a significant reduction in blood flow signal by 65 ± 8% compared to single treatments of USMB (20 ± 21%) or radiation (20 ± 32%) given alone.^20^ However, in the work here, combining USMB with HT did indeed result in a significant reduction in blood flow, a similar pattern of flow reduction was also observed with a single treatment of HT.

Photoacoustic imaging is another non‐invasive treatment modality that can be used to measure total hemoglobin and oxygen saturation level.^12^, ^24^ The results here demonstrated a significant reduction in oxygen saturation with a combined treatment of USMB + 50 minutes HT exposed groups compared to USMB‐only treatment. However, no significant effect on oxygen saturation was observed with treatments that included 10 minutes HT‐only, 50 minutes HT‐only or combined treatment of USMB + 10 minutes HT or USMB + 50 minutes HT compared to control. It has been demonstrated earlier that upon combining USMB with radiation therapy, oxygen saturation tends to decline in parallel to blood flow diminishment.^12^ However, in this study, no such correlation was found. Furthermore, 10 minutes HT‐alone treatment or the combination of USMB + 10 minutes HT showed an obvious trend of increase in oxygen saturation compared to control, however, it was not significant. This could likely be a transient change upon the initial heating duration or might be due to animal variability. It should be pointed out that the 50 minutes HT treatment either alone or combined with USMB showed oxygen saturation changes in a similar pattern to that of control groups.

Several studies regarding tumor blood flow and oxygen saturation have been previously reported combining USMB with radiation,^12^, ^20^, ^24^, ^25^, ^26^ however very few attempts have been reported with the combination of USMB and HT. Unlike radiation which causes a continuous decrement in tumor blood flow and oxygen content with increasing radiation doses, HT shows a variation in responses. Specifically, upon localized HT exposure, blood flow in normal tissue is markedly increased whereas, in tumors, the blood flow seems to follow two different flow patterns: flow may increase transiently followed by a quick decline or flow may decrease continuously with an increase in heating time and or temperature.^27^ Generally, moderate HT (41–43°C) with a short duration of heating induces an increase in tumor blood flow further resulting in increased tumor oxygen content.^11^ Moreover, the changes in tumor oxygenation levels during HT are closely associated with a rise or fall of blood flow levels.^28^ The effect of HT on tumor blood flow and tumor oxygenation has been periodically reported in past.^11^, ^29^ Those studies suggested that the amount of heat applied as well as the duration of heating were the main factors for an increase or decrease of tumor blood flow and oxygenation level. A study performed by Bicher et al reported that upon heating at 41°C, an increase in tumor blood flow with an increase in oxygen tension was observed in C3H mouse mammary adenocarcinoma. A reverse phenomenon with reduced blood flow and oxygen content was noted when the temperature was lowered below 41°C.^28^ Similarly, the duration of heating is also known to induce different blood flow patterns. Vaupel et al reported that when heated at 43°C for 20 minutes or 44°C for 15 minutes, 60% of rats DS carcinoma tumors resulted in a significant increase in blood flow whereas when the heating time was prolonged further blood flow was reduced.^30^ Moreover, a study by Stewart and Begg reported that mouse fibrosarcoma tumors, when heated at 42.5°C for 30 minutes, resulted in a significant increase in blood flow whereas with further heating duration by the end of 1‐hour blood flow was drastically reduced reaching the level of control.^31^

The findings here demonstrated a reduction in tumor blood flow already starting with 10 minutes HT treatment alone that prolonged evident also with 50 minutes treatment. Meanwhile, oxygen saturation showed an incrementing pattern with the initial 10 minutes of heating then showing a drop at 50 minutes HT treatment. The reason a small increase in oxygen saturation at 10 minutes heating time compared to control was observed is still unclear despite the fact that tumor blood flow affects oxygen saturation directly.^11^ However, the combined treatment of USMB with 50 minutes HT resulted in a significant reduction in blood flow as well as oxygenation level comparing USMB‐only treated animals.

In order to support the findings obtained from power Doppler and Photoacoustic imaging data, an immunohistological analysis of cell death and vessel count/labeling in tumors was performed. Changes in cell death and vessel count were investigated using TUNEL and CD31 staining, respectively. Results demonstrated a significant increase in cell death throughout different treatments including 10 minutes HT alone, 50 minutes HT alone, USMB + 10 minutes HT, USMB + 50 minutes HT compared to control. Similarly, compared to USMB‐only treated animals, treatment with 50 minutes HT alone or combined USMB + 50 minutes HT demonstrated a significant elevation in cell death level. Moreover, a similar trend but with a reduction in CD31 labeling was observed with different treatments of 10 minutes HT alone, 50 minutes HT alone, USMB + 10 minutes HT, USMB + 50 minutes HT compared to control. Further, comparing USMB‐only treated animals to 50 minutes HT alone or combined USMB + 50 minutes HT indicated a significant reduction in a vessel count. The bulk of evidence on tumor cell death response and vascular disruption upon USMB exposure has been reported recently. Under certain conditions treatment with USMB causes endothelial cell membrane perturbations leading to a pronounced vascular disruption followed by an enhancement of tumor cell death when combined with radiation. The findings have been validated using different tumor xenograft models including bladder, breast, prostate cancer, etc..^23^, ^32^, ^33^

Several studies have been reported demonstrating an existing correlation between tumor blood flow and oxygen saturation with cell death and vascular disruption.^12^, ^20^ Czarnota and the group have investigated the effects of USMB with radiotherapy on tumor vasculature as well as tumor cell death responses. A study carried out by Briggs et al in prostate cancer xenografts demonstrated a combination of USMB with 8 Gy resulted in a significant reduction in tumor oxygenation level as well as blood flow by 28 ± 10% and 44 ± 9%, respectively. Furthermore, the findings were found to be correlated with an increase in tumor cell death by 31 ± 5%, with a subsequent reduction in intact vasculature by 15 ± 2%.^12^ Moreover, similar outcomes were reported by El Kaffas et al using MCA‐129 fibrosarcoma xenografted tumor, where the effect of USMB with a radiation dose of 2 or 8 Gy was studied. The study demonstrated a 46% decrease in the vascular index corresponding to blood flow signal observed within 3 hours persisting from 24 to 72 hours with combined treatment of USMB + 2 Gy. The effect was further accompanied by extensive tumor cell death.^25^

In this study, the work was carried out in order to determine if similar synergy takes place combining USMB with moderate HT at 43°C. The results from power Doppler, TUNEL, and CD31 analysis revealed significant effect on tumor blood flow, cell death, and vascular disruption with as early as 10 minutes of HT treatment. The exact mechanism of HT effect at an early duration of 10 minutes is still obscure but it might be due to the sensitivity of this specific tumor xenograft type upon heating. The photoacoustic data here revealed a significant effect on oxygenation with the combination treatment only when comparing USMB and 50 minutes HT to USMB‐only treated groups.

In conclusion, the study here demonstrated USMB and HT did not act synergistically but both alone and together demonstrated a significant impact on tumor vascular damage. Future work should include the sequencing of HT and USMB as well as optimization of the time interval between these two treatment modalities. When using HT as a cancer treatment modality, precise temperature and heating time are of utmost importance. A slight increase in HT temperature and duration might cause damage to normal cells or tissue whereas inadequate heating may sometimes be unfruitful. Several clinical studies have incorporated different heating temperatures and timing to treat various cancers. A total HT duration of 30–60 minutes is used to treat tumor locally and a regional treatment requires 30–90 minutes. On the other hand, a duration of 60–120 minutes is required for whole‐body treatment. This treatment is given in combination with either radiotherapy or chemotherapy in multiple sessions. A range between 50 and 100 minutes given at one session causes thermal ablation. The summary of exact treatment temperature and timing for specific tumors type is reviewed elsewhere.^4^

Another important aspect that needs further exploration is the exact time gap between USMB and HT. Evidence has shown that when the heat is applied within 10 minutes after radiation treatment there is a greater effect on tumor cell kill.^34^ Modulating the time interval between USMB and HT could then produce different results. In this study, a 5 hour time interval between USMB and HT treatment was chosen based on our other work showing a maximal ceramide (apoptotic molecule)‐effect 6 hours after USMB treatment.^20^ Ceramide is reported to peak between 4 and 6 hours following treatments.^35^ So, we anticipated that USMB induced ceramide generation would sensitize the cells during HT treatment exhibiting greater effect. However, no significant effect between HT only and USMB combined with HT was observed in this study. It is worth noting that breast cancer models generally have inherent vascular regeneration properties.^36^ Thus, there may be some normalization of vasculature which occurs during this 5‐hour window letting the HT effect dominate subsequently.

The involvement of ceramide in USMB and HT‐induced effects needs further exploration. Ceramide is known to play an important role in the cell death‐modulating pathway. Czarnota and groups have thoroughly studied the effect of USMB‐induced ceramide generation using several in vitro and in vivo models.^20^, ^24^, ^26^, ^32^, ^37^ Experiments using prostate xenograft demonstrated a significant increase in ceramide release causing 10‐fold higher cell death following USMB and radiation. On contrary, using ceramide, antagonists sphingosine‐1‐phosphate (S1P) prevented cell death by inhibiting the process of ceramide generation.^20^ Studies have also shown that exposure to HT can cause an increase in ceramide production. Experiments conducted with leukemic T‐lymphocytes and myeloid cell lines upon treatment with heat (1 hour at 42°C) combined with tumor necrosis factor (TNF)‐related apoptosis‐inducing ligand (TRAIL) cause a twofold increase in the level of ceramide. A rise in ceramide content resulted in significant cell kill. The process of cell death was inhibited using a ceramide inhibitor.^38^ Thus, these data suggest that both USMB and HT can separately induce ceramide generation on its own therefore we hypothesized that using both these two modalities together might enhance the ceramide content within the tumor augmenting the treatment effect. Furthermore, lowering the thermal doses of HT might provide a clearer understanding as greater distinguishable effects on tumor cell death and vascular disruption would be observed. Further experimental studies are necessary to evaluate these hypotheses.

## Supporting information


**Supplemental Figure 1** Diagram of the experimental setup for water bath hyperthermia. The temperature of the water bath was kept 43°C. Custom‐made tubes were used to mount the animals in a way that only the tumor‐bearing leg was submerged in the water for treatment. The tube consisted of several holes to act as an air vent with an additional hole in the lid opening for the leg and tail to pass through it. The tubes were attached to the customized plate using Velcro attachments. The tube‐attached plate was submerged in the water bath that was supported by a 3‐prong extension clamp attached to a support stand. A thermometer was affixed to the tube‐attached plate for constant monitoring of temperature.
