# Supplementary figures and images for: Effect of Ultrasound‐Stimulated Microbubbles and Hyperthermia on Tumor Vasculature of Breast Cancer Xenograft
Source: J Ultrasound Med. 2022 Feb 10;41(11):2659–71. doi: 10.1002/jum.15950 (PMC9790356; doi:10.1002/jum.15950)

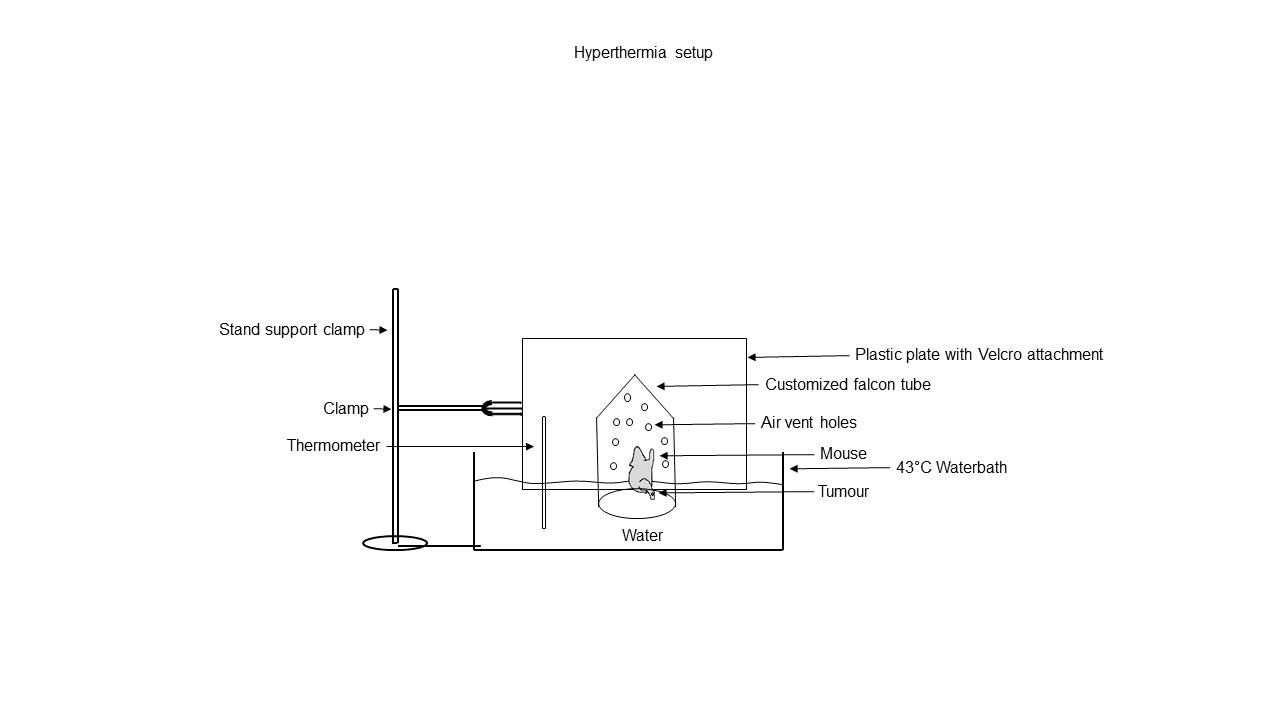

Supplement: Supplementary file 1 — Supplemental Figure 1 Diagram of the experimental setup for water bath hyperthermia. The temperature of the water bath was kept 43°C. Custom‐made tubes were used to mount the animals in a way that only the tumor‐bearing leg was submerged in the water for treatment. The tube consisted of several holes to act as an air vent with an additional hole in the lid opening for the leg and tail to pass through it. The tubes were attached to the customized plate using Velcro attachments. The tube‐attached plate was submerged in the water bath that was supported by a 3‐prong extension clamp attached to a support stand. A thermometer was affixed to the tube‐attached plate for constant monitoring of temperature. [file JUM-41-2659-s001.tif]
